# Case Report: Adrenal glands degenerated schwannoma: Report of three cases and literature review

**DOI:** 10.3389/fonc.2023.990028

**Published:** 2023-01-23

**Authors:** Tao Zhang, Si-fan Yin, Wen-bo Feng, Run-lin Feng, Chang-xing Ke

**Affiliations:** ^1^ Department of Urology, The Second Affiliated Hospital of Kunming Medical University, Kunming, China; ^2^ Department of Pathology, The Second Affiliated Hospital of Kunming Medical University, Kunming, China

**Keywords:** diagnosis, treatment, clinical features, adrenal glands, degenerated schwannoma

## Abstract

**Background:**

Schwannoma is a benign tumor, of which degenerated schwannoma is a subtype. Retroperitoneal schwannomas are extremely rare, as they account for only 3% of retroperitoneal tumors.Degenerated schwannoma is a schwannoma subtype. However,degenerated schwannoma occurring in the adrenal glands is extremely rare.

**Case summary:**

Case 1: A 42-year-old man was referred to our hospital for further examination of a left adrenal mass that was incidentally discovered during a routine physical check-up.No significant abnormalities were found in laboratory tests results. Robotic-assisted laparoscopic excision of the left adrenal gland was performed under general anesthesia. Case 2: A 47-year-old man was admitted to the hospital because of a left adrenal mass found on a routine physical examination.The patient was previously in good health, and there was no family history of a similar disorder. Left-sided laparoscopic adrenalectomy was performed under general anaesthesia. Case 3: A 62-year-old woman with hypertension and diabetes mellitus was referred to our hospital after an incidentally found left adrenal mass.There was no family history of a similar disorder. Left-sided laparoscopic adrenalectomy was performed under general anaesthesia. None of the patients had a recurrence in our study during the postoperative follow-up.

**Conclusion:**

Degenerated schwannoma of the adrenal glands is very rare. The clinical presentations of degenerated schwannoma are nonspecific; a small number of patients do not have any symptoms, and the mass is only found incidentally during physical examination for any number of reasons. The preoperative diagnosis of adrenal degenerated schwannoma is difficult because the diagnosis must rely on pathological examination and immunohistochemistry assays. The management is surgical excision and regular follow-up.

## Introduction

Schwannomas are composed of Schwann cells arising from a peripheral nerve sheath. It is a benign tumor. Degenerated schwannoma is a subtype of schwannoma (1, 2). Degenerated schwannoma in the adrenal gland is extremely rare. Pathogenesis is still unclear, and clinical presentation is not specific. We summarized the clinical and pathological data of patients pathologically diagnosed with degenerated schwannoma in the adrenal glands to improve the understanding of this disease entity.

### Case introduction

Three cases of degenerated schwannoma were diagnosed in patients between 26 and 62 years of age (1 females and 2 male).

#### Case 1

A 26-year-old man was evaluated at the hospital for a left adrenal mass that had been identified incidentally during a physical examination 5 months prior.No significant abnormalities were found in laboratory tests results. Imaging for this case was done at an outside hospital and is no longer available.Robot-assisted laparoscopic excision of the left adrenal gland was performed under general anaesthesia. On gross examination, the specimen was a greyish white and greyish red cyst, measuring 5.0 cm x 3.0 cm x 1.0 cm. Immunohistochemistry showed:VIM (+), S100 (+), ki-67 (10%), SOX10 (+), CD34 (vascular +). The pathologic examination led to a final diagnosis of adrenal glands degenerated schwannoma. No recurrence was observed during the 15 months of follow-up (**Supplementary Figure 1**).

#### Case 2

A 47-year-old man was admitted to the hospital because of a left adrenal mass found on a routine physical examination.The patient was previously in good health and had no family history of a special disease. The patient was in good general conditions, physical examination and neurodevelopmental milestones were normal by his age. Systemic examination revealed no abnormalities.The plasma normetanephrine levels were normal. The preoperative plasma renin levels were in the normal range in the supine (2.62 pg/mL; reference ranges 4-24) and upright positions (3.69 pg/mL; reference ranges 4-38). The ultrasound showed a hypoechoic area in the left adrenal gland, measuring approximately 4.2 cm in the right-left diameter × 2.8 cm in anterior-posterior diameter, it had clear borders and a regular morphology without apparent internal blood flow signals. The plain CT scan and contrast-enhanced CT scan revealed a a left adrenal mass, measuring approximately 4.0 cm in the right-left diameter × 2.9 cm in anterior-posterior diameter. A laparoscopic left adrenalectomy was performed under general anesthesia. Gross specimen showed that the capsule was intact with multiple incisions, and the cut surface appeared to be greyish grey-brown with congestion and necrosis. Immunohistochemistry showed:S100 (+), ki-67 (1%), SMA (-), CD56 (+), CD31 (vascular +). The pathologic examination led to a final diagnosis of adrenal glands degenerated schwannoma. No recurrence was observed during the 40 months of follow-up (**Supplementary Figure 2**).

#### Case 3

**Figure 1:**
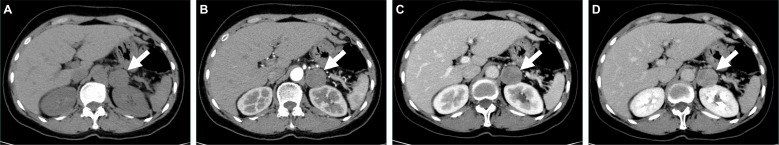
**(A–D)** CT of the patient before surgery.

**Figure 2:**
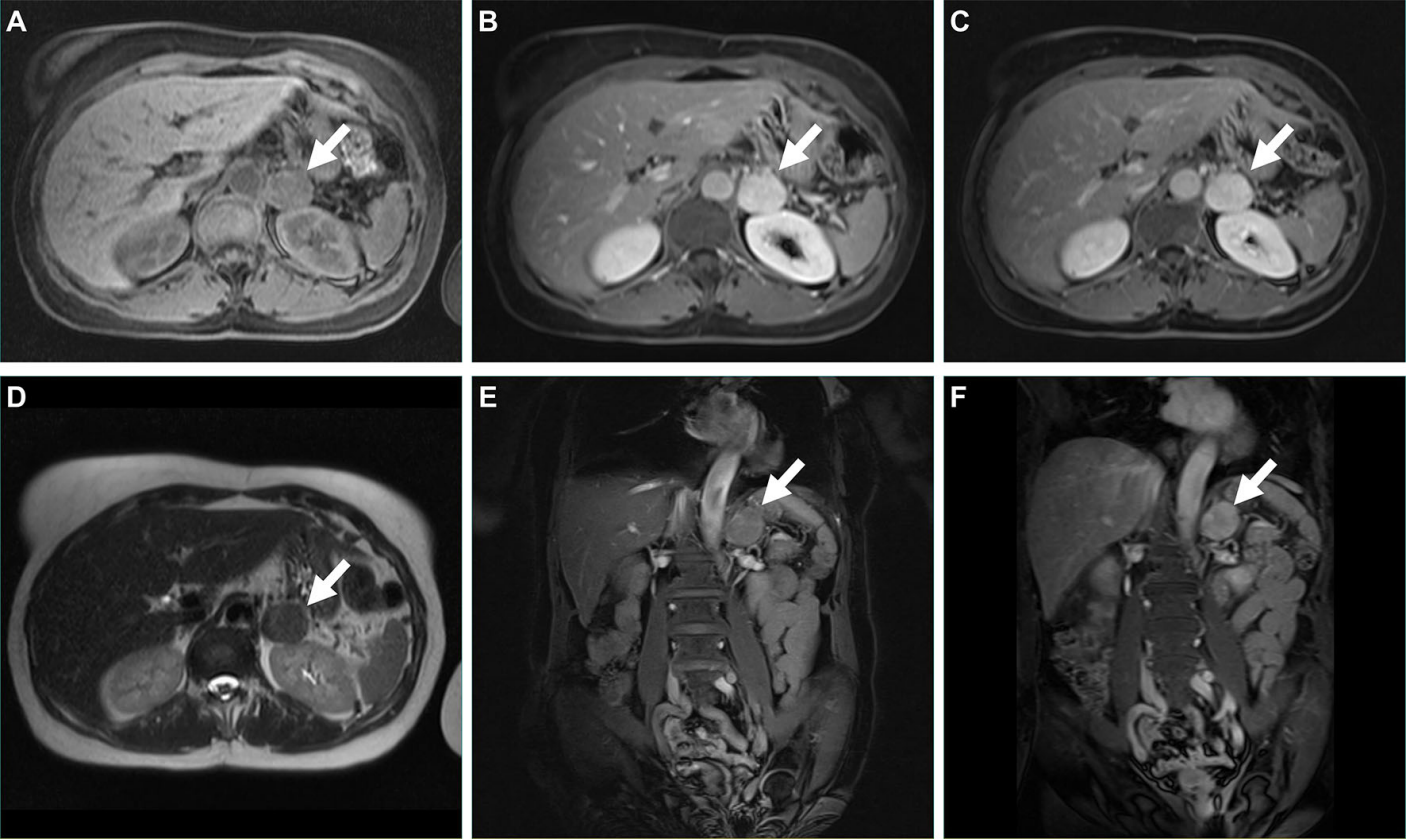
**(A–F)** MRI of the patient before surgery.

**Figure 3:**
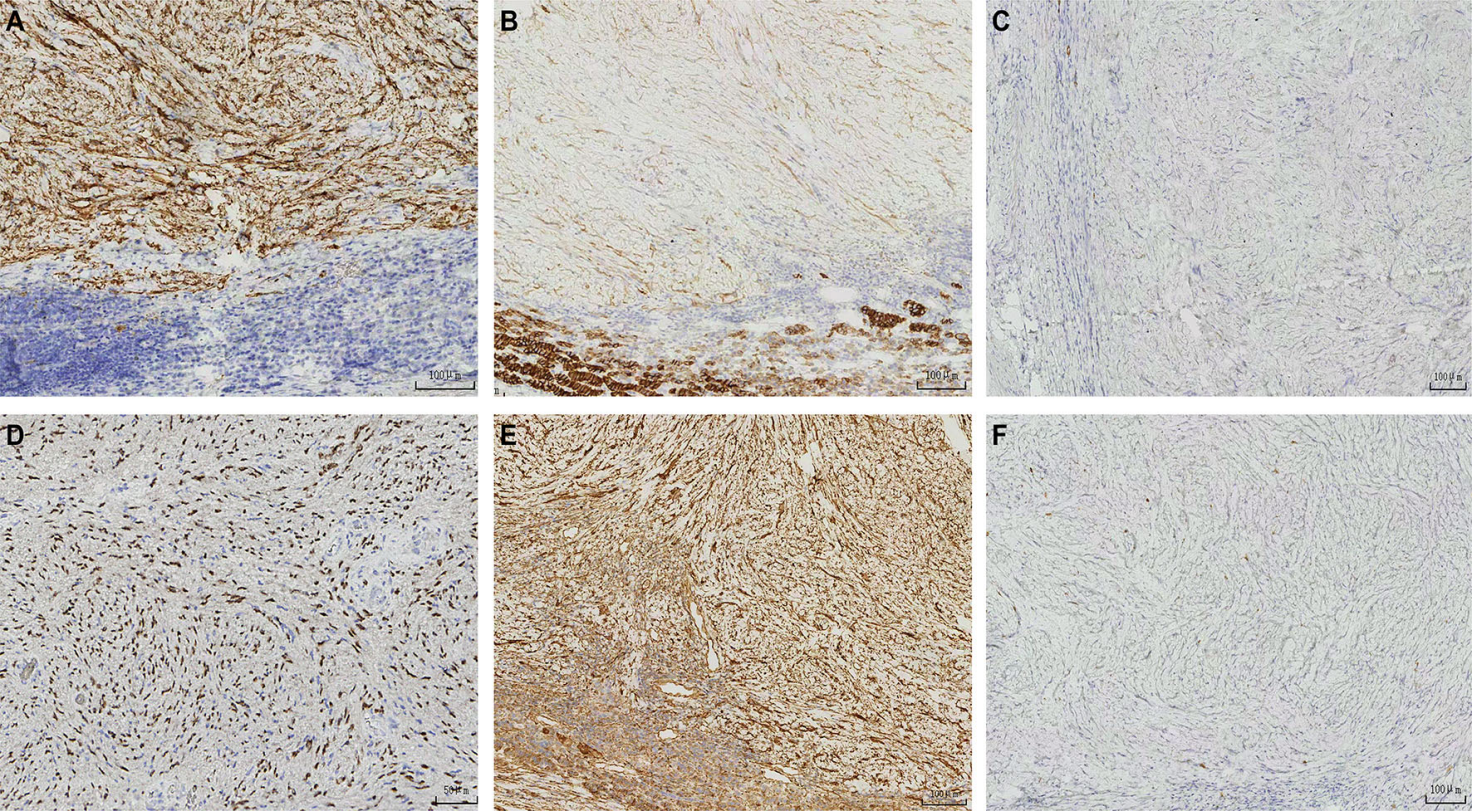
**(A–F)** The graph of immunohistochemical staining.

A 62-year-old woman with hypertension and diabetes mellitus was referred to our hospital because of an incidentally found left adrenal mass. There was no family history of a similar disease. On physical examination, the patient was in good general condition, pleuropulmonary and abdominal examination was normal. The plain CT scan and contrast-enhanced CT scan showed a nodular lesion in the left adrenal gland, measuring approximately 3.4 cm in the right-left diameter × 3.0 cm in anterior-posterior diameter, with heterogeneous enhancement (**Figure 1**). MRI scan + enhancement showed a rounded, well-defined nodule in the left adrenal gland, measuring approximately 3.2 cm in the right-left diameter × 2.7 cm in anterior-posterior diameter × 3.3 cm maximal superior-inferior diameter, with Delayed and progressive enhancement (**Figure 2**). A laparoscopic left adrenalectomy was performed under general anaesthesia. Gross specimen showed an intact capsule with a greyish white cut surface. Postoperative examination showed a degenerated Schwannoma (left adrenal gland). S100 (+) (**Figure 3A**), CD56(+) (**Figure 3B**), CKL(-) (**Figure 3C**), SOX-10(+) (**Figure 3D**),VIM (+) (**Figure 3E**), ki-67 (3%) (**Figure 3F**), SMA (-), CD34 (vascular +). The pathologic examination led to a final diagnosis of adrenal glands degenerated schwannoma. No recurrence was observed during the 33 months of follow-up (**Supplementary Figure 3**).

## Related literature learning

Schwannoma (Neurilemmoma) is a benign tumor of nerve sheath origin. It contains multiple subtypes. For example, classic schwannoma, degenerated schwannoma, cellular schwannoma, plexiform schwannoma, etc (1, 3). Schwannomas usually arise in the spinal nerve roots, intracranial nerves, and extremities (4). It is more common in women between 20-50 years-old. Schwannoma mostly occurs in intracranial, cervical, thoracic, or deep soft tissue lesions (4). Schwannomas rarely occur in the retroperitoneum, accounting for only 3% of retroperitoneal tumors. Cellular schwannoma has similar sites to schwannoma but tends to develop more often in deep structures, especially in the posterior mediastinum and paraspinal region. Plexiform schwannoma usually occurs in the superficial soft tissues of the head and neck. Deep-seated nerves are rarely affected. As a subtype of schwannoma, degenerated schwannoma, also known as ancient schwannoma (ancient is assumed to be a state of aging), was first reported in 1951 (5). It is characterized by calcification and cystic degeneration (6). Cellular proliferation, cellular anisotropy, and degenerative changes may be seen. The pathogenesis of degenerated schwannoma is unknown. It may be related to inflammation or mechanical stimulation such as irritation, or it may have no apparent cause (1, 2). Plexiform schwannoma has similar characteristics to the other types but is more likely to progress to malignancy. It is typically characterized by a plexiform or multinodular growth pattern. Cellular schwannoma is often a single mass, but can also be multiple.

Degenerated schwannoma arising from the adrenal glands is an extremely rare entity (7). The clinical presentation of degenerated schwannoma, cellular schwannoma, and plexiform schwannoma is usually asymptomatic, but they can present with nonspecific signs and symptoms The tumor is typically asymptomatic in the retroperitoneum. The retroperitoneum is a large space where degenerated schwannoma grows silently before clinical signs appear (7, 8). This was consistent with previous reports in the literature.

In gross specimen, the tumor had a complete capsule with a well-defined border. In case 1, a greyish white and greyish red cyst with a localised greyish brown cauliflower-like projection on the inner wall of the cyst. In case 2, intact pericardium, multi-sectional incision, greyish grey-brown surface appears to be congested and necrotic. In case 3, complete envelope, greyish white cut surface. Based on previous reports and the present case, it is assumed that most of the degenerated schwannoma in the adrenal gland have intact envelope with well-defined margin and greyish white cut surface.

**Figure 4:**
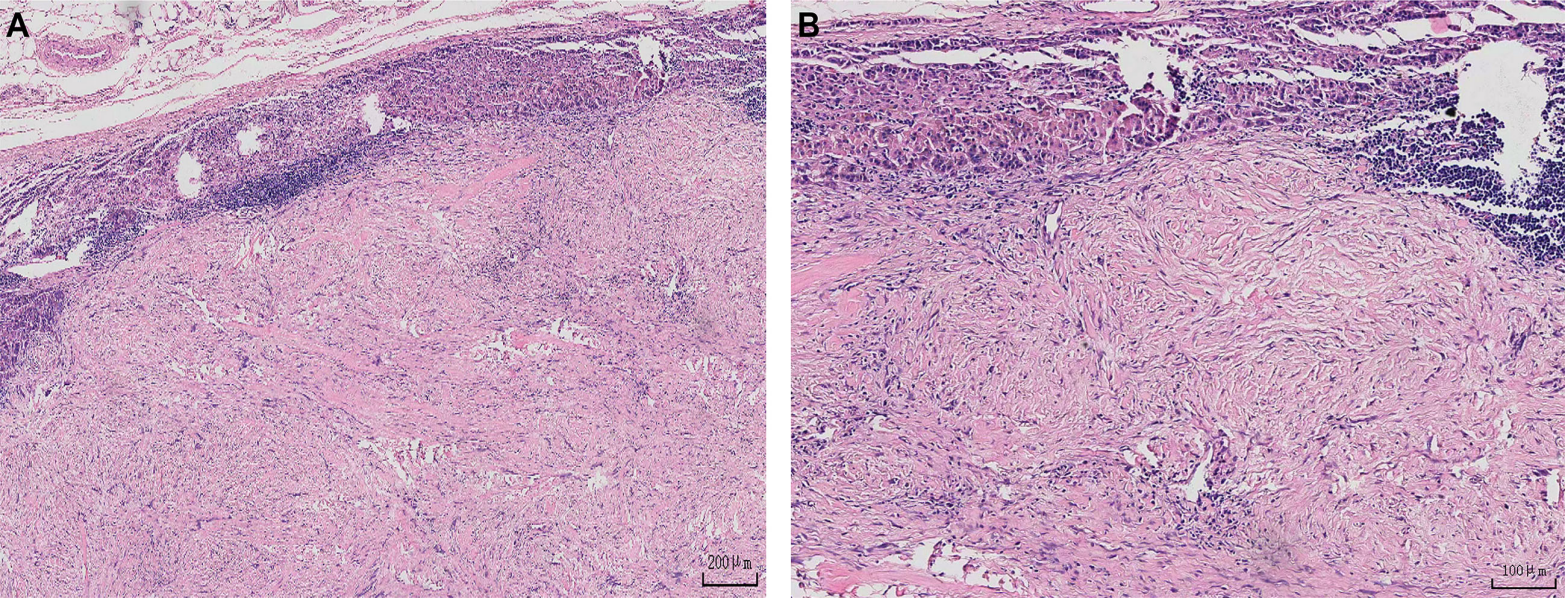
**(A, B)** A Diagram of the Pathology.

Degenerated schwannoma is benign tumor. It has a high cell count and cellular anisotropy. Multiple components can be observed: hemorrhage, mucoid degeneration, calcification, inflammatory cell infiltration, hyaline changes in the vessel wall, cystic lumen formation, and nuclear pleomorphism. Histologically, schwannoma is composed of two different types (Antoni A and B). Antoni A type shows mainly densely packed, spindle-shaped cells arranged in short, interwoven bundles and whorls. Antoni B type has a looser structure with cystic spaces mixed within the tissue. Degenerated schwannoma often shows a decrease in Antoni A type. It is mostly presented as the Antonian B type. Degenerative changes are often associated with cellular heterogeneity. It can be easily misdiagnosed as a malignant neoplasm. These two patterns may coexist, but usually, one is predominant in cellular schwannoma. Plexiform schwannoma may not always display the Antoni A and B patterns (but when it does, Antoni A is predominant). S-100 immunohistochemistry analyses reveal positive staining for this marker, which supports the diagnosis of schwannoma (2). In this group, the Antoni A type was reduced and most of them showed the Antoni B type (**Figure 4**). In this group of cases, S100 was positively expressed and the ki-67 index was low, which indicated a low degree of malignancy and slow cell proliferation.

Imaging features are usually nonspecific. CT-guided fine needle aspiration biopsy does not appear to provide an accurate preoperative diagnosis. It is easy to diagnose malignant tumors. It is not recommended to carry out puncture biopsy (7, 8). The MRI indicates a benign schwannoma with smooth margins. Compared to muscle, it is isosignal on T1WI and has a high signal on T2WI (8). It was difficult to speculate on the imaging manifestations of this group of cases because there were few relevant imaging examinations. There is no characteristic of an imaging examination for the diagnosis of cellular schwannoma and plexiform schwannoma.Definitive diagnosis still relies on postoperative histopathological examination and immunohistochemistry.We need to be differentiated from the following diseases:1) Pleomorphic sarcoma: It is also known as malignant fibrous histiocytoma. Immunohistochemical markers lack specificity. Pleomorphic sarcoma is one of the most common soft tissue sarcomas, representing 28% of all soft tissue sarcomas (9, 10). Its occurrence is associated with radiotherapy, long-term chronic irritation, and lymphedema. However, the cause of the disease is unknown. Pleomorphic sarcoma mainly occurs in middle-aged and elderly people. It is most often seen in the extremities and retroperitoneum. Local invasion and distant metastasis can occur. We can see the secretion of catecholamines and cortisol (10). Microscopically, the histological characteristics of pleomorphic sarcoma include the complexity of cell components, pleomorphism of tumor cells, and the diversity of tissue structure (11). Immunohistochemistry shows positive staining with waveform protein, actin, CD68, alpha1-chymotrypsin and alpha-1-antichymotrypsin. Surgical treatment is preferred therapy. The prognosis is poor (12). 2) Mucinous liposarcoma: Mucinous liposarcoma is a subtype of liposarcoma. It can easily metastasize outside the lungs. The most common metastases occur in the retroperitoneum. Whole-body MRI is superior to CT for diagnosing bone metastases. CT can only detect 14% of patients with bone metastases (13, 14). The sensitivity of x-rays and bone imaging is even worse. 3)Malignant schwannoma: It is a rare and highly aggressive form of the disease. It occurs mostly in the extremities, chest wall, and abdominal wall. Laryngitis can also occur. Malignant schwannoma accounts for 5%-10% of all soft tissue tumors. It can be induced by radiotherapy. It can also be derived from the presence of a previous associated neoplastic lesion. The clinical presentation is non-specific. It is characterized by “marble-like” spindle-shaped tumor cells. Malignant schwannoma is a highly malignant sarcoma. Malignant schwannoma has a high risk of local recurrence and distant metastasis. Survival rate is generally low (8, 15).

Malignant transformation may rarely occur. During this long period in which tumor volume did not change, it is feasible to observe it. Surgery remains the primary treatment modality, with complete laparoscopic resection of the tumor being preferred (8, 16). After surgical excision of the lesion recurrence is rare (16). It is possible to treat such patients with radiotherapy to reduce blood supply and allow resection (16). Complete resection of the tumor was performed in three cases.No recurrence was seen in any of the patients. There are some limitations and shortcomings in this study. For example, it’s a retrospective study and the number of cases is small. Future multicenter studies with a larger sample size can provide more accurate information about the adrenal glands’ degenerated schwannoma.

## Conclusion

In conclusion, the pathogenesis of adrenal glands degenerated schwannoma is unknown.The clinical presentation is non-specific. The diagnosis depends on pathology and immunohistochemistry, with positive expression of S100 in immunohistochemical markers.

## Data availability statement

The original contributions presented in the study are included in the article/supplementary material. Further inquiries can be directed to the corresponding authors.

## Ethics statement

The patients/participants provided their written informed consent to participate in this study. Written informed consent was obtained from the individual(s) for the publication of any potentially identifiable images or data included in this article.

## Author contributions

TZ, S-FY and R-LF collected data and drafted the manuscript. R-LF provided pathology results and drafted the manuscript. W-BF collected data and reviewed the manuscript. C-XK edited the manuscript and critically revised the draft. All authors contributed to the article and approved the submitted version.

## References

[1] Yasuhide Takeuchi YA Yokoo H Mikami Y Terada Y Yoshida K Miyamoto S . Intra-cerebellar schwannoma with various degenerative changes: A case report and a systematic review. BMC Neurol (2022) 66. doi: 10.1186/s12883-022-02596-3 PMC8867888 35209854

[2] Argenyi ZB Balogh K Abraham AA . Degenerative (“ancient”) changes in benign cutaneous schwannoma. A light microscopic, histochemical and immunohistochemical study. J Cutan Pathol (1993) 20(2):148–53. doi: 10.1111/j.1600-0560.1993.tb00232.x 8320360

[3] Bore P Descourt R Ollivier L Le Roux PY Abgral R . False positive 18f-fdg positron emission tomography findings in schwannoma-a caution for reporting physicians. Front Med (Lausanne) (2018) 5:275. doi: 10.3389/fmed.2018.00275 30349818 PMC6186987

[4] Noh S Do JE Park JM Jee H Oh SH . Cutaneous schwannoma presented as a pedunculated protruding mass. Ann Dermatol (2011) 23(Suppl 2):S264–6. doi: 10.5021/ad.2011.23.S2.S264 PMC3229082 22148067

[5] Choudry HA Nikfarjam M Liang JJ Kimchi ET Conter R Gusani NJ . Diagnosis and management of retroperitoneal ancient schwannomas. World J Surg Oncol (2009) 7:12. doi: 10.1186/1477-7819-7-12 19187535 PMC2645401

[6] Gulati V Swarup MS Kumar J . Solid primary retroperitoneal masses in adults: An imaging approach. Indian J Radiol Imaging (2022) 32(2):235–52. doi: 10.1055/s-0042-1744142 PMC9340194 35924125

[7] David Pinto OK-P Cho M Zundel N Szomstein S . Rosenthal RJ . Laparoscopic resection of a retroperitoneal degenerative schwannoma: A case report and review of the literature. Surg Laparosc Endosc Percutan Tech (2008) 18:121–3. doi: 10.1097/SLE.0b013e3181581fab 18288004

[8] Daneshmand S Youssefzadeh D Chamie K Boswell W Wu N Stein JP . Benign retroperitoneal schwannoma: A case series and review of the literature. Urology (2003) 62(6):993–7. doi: 10.1016/s0090-4295(03)00792-1 14665342

[9] Fuu T Yano A Urakami S . Undifferentiated pleomorphic sarcoma of the retroperitoneum mimicking a cortisol- and catecholamine-secreting adrenal tumor. IJU Case Rep (2022) 5(3):195–8. doi: 10.1002/iju5.12436 PMC9057752 35509781

[10] Han X Zhao L Mu Y Liu G Zhao G He H . Undifferentiated high-grade pleomorphic sarcoma of the colon: A rare case report and literature review. BMC Gastroenterol (2022) 22(1):115. doi: 10.1186/s12876-022-02189-x 35272624 PMC8908612

[11] Tetteroo B . Malignant fibrous histiocytoma of the sigmoid: A case report and review of the literature. Int J Colorectal Dis (2006) 22:549–52. doi: 10.1007/s00384-006-0162-1 16896996

[12] Ylermi Soini HA-H . Tumor cells of malignant fibrous histiocytomas express mrna for laminin. Am Jounal Pathol (1991) 139:1061–8. PMC1886341 1659202

[13] Oh KY Hong SD . Myxoid liposarcoma metastatic to the mandible: First case report and review of the literature. Oral Oncol (2022) 129:105900. doi: 10.1016/j.oraloncology.2022.105900 35525204

[14] Visgauss JD Wilson DA Perrin DL Colglazier R French R Mattei JC . Staging and surveillance of myxoid liposarcoma: Follow-up assessment and the metastatic pattern of 169 patients suggests inadequacy of current practice standards. Ann Surg Oncol (2021) 28(12):7903–11. doi: 10.1245/s10434-021-10091-1 33961173

[15] Schuch LF Kirschnick LB de Arruda JAA Klein IP Silveira FM Vasconcelos ACU . Malignant peripheral nerve sheath tumour of the oral and maxillofacial region-a systematic review. Oral Dis (2022) 28(8):2072–82. doi: 10.1111/odi.13982 34333825

[16] James F Regan GLJ Karl J . Retroperitoneal neurilemoma. Am J Surg (1977) 134:140–5. Schmulzer. doi: 10.1016/0002-9610(77)90297-5 879406

