# Supplementary figures and images for: Case Report: Adrenal glands degenerated schwannoma: Report of three cases and literature review
Source: Front Oncol. 2023 Jan 23;13:990028. doi: 10.3389/fonc.2023.990028 (PMC9899977; doi:10.3389/fonc.2023.990028)

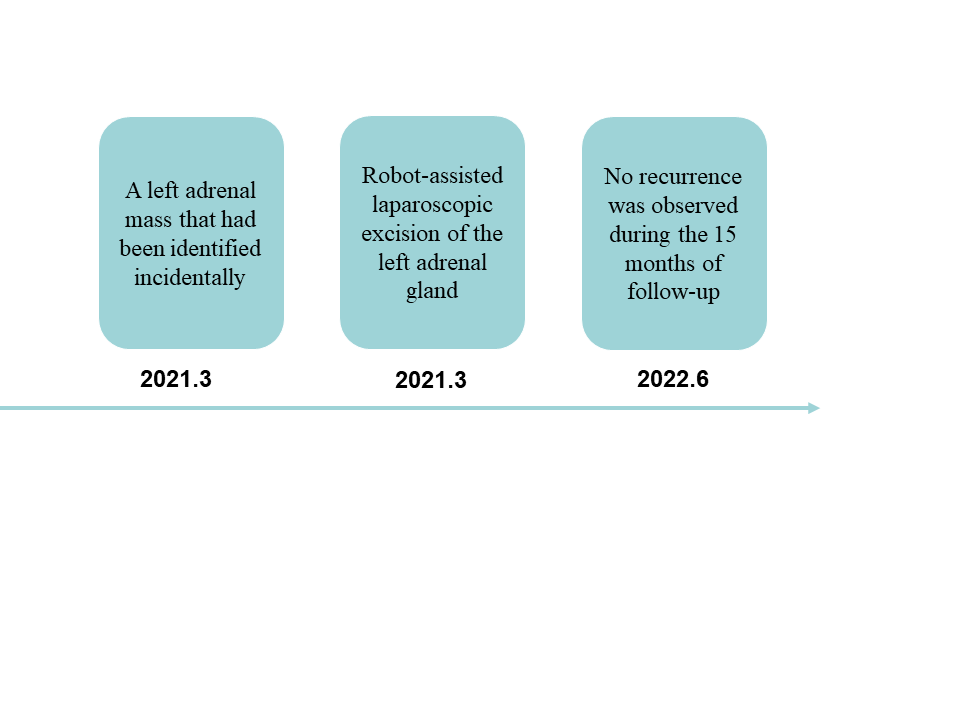

Supplement: Supplementary file 1 [file Image_1.tif]

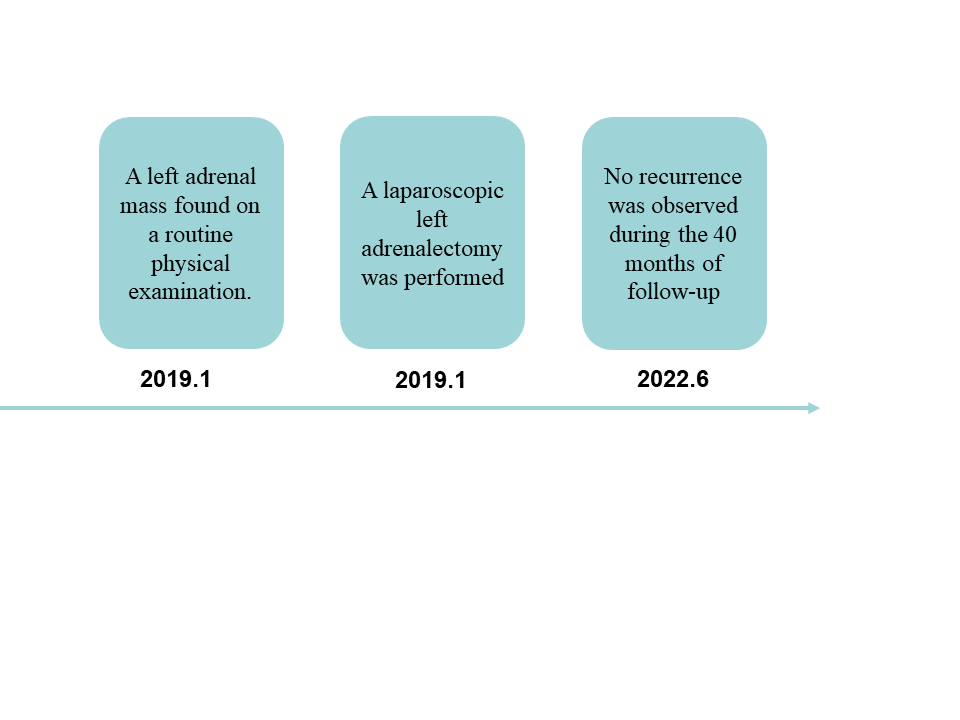

Supplement: Supplementary file 2 [file Image_2.tif]

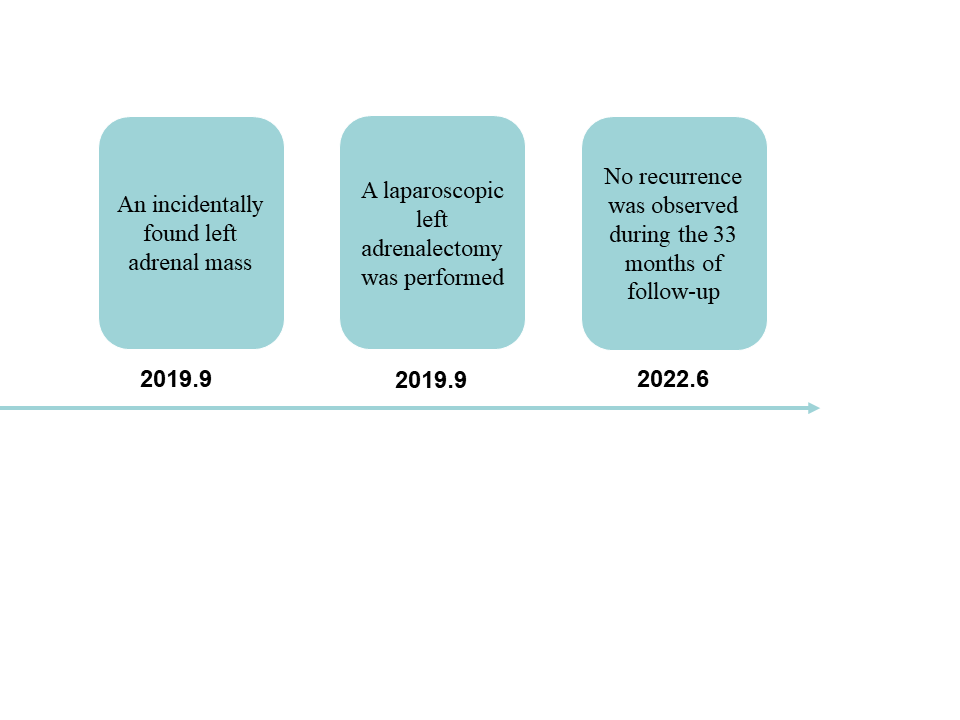

Supplement: Supplementary file 3 [file Image_3.tif]
